# Dysmenorrhea in adolescents requires careful investigation of endometriosis—an analysis of early menstrual experiences in a large case-control study

**DOI:** 10.3389/frph.2023.1121515

**Published:** 2023-08-25

**Authors:** Samia El-Hadad, Daksha Lässer, Maike-Katja Sachs, Alexandra Sabrina Kohl Schwartz, Felix Haeberlin, Stephanie von Orelli, Markus Eberhard, Brigitte Leeners

**Affiliations:** ^1^ Department of Reproductive Endocrinology, University Hospital Zurich, Zurich, Switzerland; ^2^ Division of Gynecological Endocrinology and Reproductive Medicine, Cantonal Hospital of Lucerne, Lucerne, Switzerland; ^3^ Department of Gynecology and Obstetrics, Canton Hospital St. Gallen, St. Gallen, Switzerland; ^4^ Department of Gynecology and Obstetrics, Triemli Hospital Zurich, Zurich, Switzerland; ^5^ Department of Gynecology and Obstetrics, Canton Hospital Schaffhausen, Schaffhausen, Switzerland

**Keywords:** adolescents, dysmenorrhea, endometriosis, maternal experiences, menarche, menstruation

## Abstract

**Introduction:**

Recent evidence shows that endometriosis, a significant cause of infertility, may already present in adolescents. Dysmenorrhea, often leading to school absences, is a key symptom of the maturing menstrual cycle but also of endometriosis. However, it is often perceived as “normal” and left untreated. In adolescents, laparoscopy, the standard procedure to diagnose endometriosis, is performed particularly cautiously. To improve reproductive health in adolescents, we evaluate associations between early menstrual experiences and endometriosis.

**Methods:**

Retrospective data on early menstrual experiences from 563 women with surgically/histologically verified endometriosis and from 563 age-matched controls were compared. Study participants were recruited in Switzerland, Germany, and Austria. Information on menstrual experiences was collected via a structured questionnaire.

**Results:**

The bivariate analysis showed that early menarche (*p* = 0.004), dysmenorrhea and negative memories of menarche (*p* < 0.001) were significantly associated with a diagnosis of endometriosis. After controlling for confounders in bivariate regression analysis occurrence of dysmenorrhea (*p* = <0.001, OR 5,74, 95% CI 3.82–7.22) especially with onset >3 years after menarche ((*p* = <0.001, OR 3.42, 95% CI 2.09–5.64) remained statistically significant predictors for diagnosis of endometriosis. Dysmenorrhea in mothers and mothers' perceived attitude towards menstruation were not associated with the occurrence of Endometriosis.

**Conclusions:**

Dysmenorrhea and onset of dysmenorrhea at menarche or several years after it are strongly associated with the development of endometriosis. As mothers perceived attitudes towards menstruation show no significant association with their daughters' experiences, physical symptoms accompanying menarche and menstrual period pain in adolescents seem to be very reliable predictors in diagnosis of endometriosis. Therefore, dysmenorrhea in adolescents requires careful investigation of possible endometriosis, especially if it does not respond to medical management.

**Clinical trials registration:**

The study was registered on ClinicalTrials.gov, identifier (NCT 02511626).

## Introduction

Endometriosis is a benign gynecological disease, characterized by endometrial-like tissue located outside the uterine cavity and associated with intensive chronic pelvic pain, fatigue, bleeding disorders, and infertility (1–3). Women with endometriosis often suffer from loss of productivity, professional limitations, relationship difficulties, psychological strain, and reduced quality of life (4, 5). About 10% of women of reproductive age present endometriosis (6, 7). In adult women with chronic pelvic pain, endometriosis is diagnosed in 44% (8) of cases; in adolescents with chronic pelvic pain, prevalences reach 64% (9). In terms of etiology, two main theories are discussed: Either fragments of endometrial tissue reach the peritoneal cavity through retrograde menstruation, adhere, and form endometrial lesions (10) or dislocated precursors, possibly resulting from abnormalities in the early development of the female genital tract, differentiate into lesions (3, 11). Sampson's theory of retrograde menstruation (10), together with studies reporting early age at menarche and short cycle length as risk factors for endometriosis (12–14), led to the concept that an exposure to menstruation is needed to develop endometriosis and induce symptoms such as dysmenorrhea (14). However, as endometriosis is a hormone-dependent disease (3, 15), hormonal changes related to puberty may trigger the development of endometrial lesions also from early endometrial fragments and result in early dysmenorrhea (11). Menarche and the first menstrual periods are frequently accompanied by dysmenorrhea, classified as primary dysmenorrhea without any defined underlying pathology (16, 17). There are multiple theories regarding the origin of endometriosis lesions, some of which imply early life factors (3, 18, 19, 20, 21). Therefore, early dysmenorrhea might be an important predicting factor for development of endometriosis. At the same time, young girls learn from their mothers how to deal with menstruation. Mothers' experiences represent a combination of physical symptoms (dysmenorrhea, hypermenorrhea, or premenstrual syndrome) and socio-cultural societal or individual convictions about menstruation, which are likely shared with daughters. Although general negative attitudes towards menstruation such as feelings of dirtiness, shame, and secrecy are declining (22), negative experiences of first menstruation are frequent and are described as a result of insufficient preparation, taboos of culture, religion and sexuality, and negativity towards menstruation passed on by mothers (23, 24). Therefore, it is difficult to distinguish to which degree dysmenorrhea is indeed the result of physical factors or the results of pain expectations, education, and shared family experiences. In the context of endometriosis, the relation between a potential influence of mothers' experiences with menstruation on their daughters' experiences is rather complicated since mothers of women with endometriosis also have an increased prevalence of endometriosis. However, no previous quantitative or qualitative studies have explored dysmenorrhea experiences in the mother-daughter dyad in the context of endometriosis. Endometriosis is prevalent, induces symptoms that severely impact not only quality of life but also the ability to fulfill daily tasks, which is particularly important during adolescence when foundations for later life are established, for example though education.

Dysmenorrhea in adolescents is one of the main factors for regular school absences and productivity loss when present at school (25). Therefore, early identification and adequate treatment are necessary for successful adolescent development. Furthermore, endometriosis is a leading cause of infertility, with a prevalence of 0.5%–5% in fertile but 25%–40% in young infertile women (8, 12). The causes of infertility in women with endometriosis may range from anatomical distortions due to adhesions and fibrosis to endocrine abnormalities and immunological disturbances. The various pathophysiological disturbances seem to interact through mechanisms so far not fully understood (12). Correct diagnosis of endometriosis in young women is crucial to allow them adequate decision making when it comes to planning initiation of family.

As accurate non-invasive diagnostic tests or biomarkers for endometriosis are not yet identified, the development of a non-invasive screening tool and the improvement of endometriosis diagnosis are listed in the “top ten research priorities for endometriosis” (26). It is hoped that a better understanding of key disease symptoms against the background of confounding factors in association with early menstruation will advance early diagnosis as an important step towards a treatment for endometriosis adjusted to the specific situation of adolescents.

Therefore, our primary aim was to evaluate early somatic (i.e., age at menarche, dysmenorrhea) and mental menstrual experiences (i.e., memories related to menarche) for their associations with the development of endometriosis, taking mothers' experiences and attitudes towards menstruation into account.

## Methods

This study was part of a multicenter case-control study investigating risk factors and characteristics of quality of life in women with endometriosis compared to control women. The primary outcome of this study was to detect associations between early menstrual experiences and diagnosis of endometriosis. A secondary outcome addressed the timing of onset of dysmenorrhea for its relevance in the development of endometriosis. The manuscript was prepared following STROBE-criteria (Strengthening the Reporting of Observational Studies in Epidemiology) (27).

### Setting and recruitment

Study participants were recruited at university hospitals, district hospitals, and associated doctor's practices in Germany, Austria, and Switzerland (1, 4, 28, 29). University Hospital Zurich, the Triemli Hospital Zurich, the district hospitals in Schaffhausen, Solothurn, St. Gallen, Winterthur, Baden, and Walenstadt, the Charité Berlin, the Vivantes Humboldt Hospital Berlin, the Albertinen Hospital Hamburg, the University Hospital Aachen, and the University Hospital Graz contributed substantially (1, 4, 28, 29). Participating women were approached directly by medical staff and informed that the study was voluntary and that only encrypted data would be processed and published. Interested women received additional written information and signed consent forms for participation as well as for verification of diagnosis in the hospital where surgery occurred. Women with endometriosis were recruited at post-operative follow-ups or check-ups regarding disease development. Control women were invited to participate at regular annual consultations or hospital stays for temporary, minor gynecological diseases/surgery. Additionally, a smaller group of women diagnosed with endometriosis (*N* = 57) was recruited from support groups for endometriosis in Germany. These women were contacted by experienced members from the German Endometriosis Society (www.endometriose-vereinigung.de).

All women were reminded after 1 month and after 3 months to return the questionnaire to achieve a higher response rate. Frequently mentioned reasons for refraining from participation were lack of time or too personal questions.

### Inclusion and exclusion criteria

The present evaluation was part of a larger study where a sample size of 387 participants in each group was calculated to detect a 10% difference between cases and controls with an alpha of 0.05 and a power of 0.8 (1, 4, 28, 29). Our final sample size included 563 women with endometriosis and 563 control women. For inclusion in the study, women had to give their written consent for participation and for confirmation of diagnosis from surgical reports. They had to show the psychological and linguistic ability to understand and answer the questionnaire. Women with endometriosis were included after surgically and histologically confirmed disease regardless of current symptoms, stage, and severity of disease. Endometriosis verification was obtained from surgical reports and classified according to rASRM (revised American Society for Reproductive Medicine) classification. Only women without pelvic pain at the time of the study or after exclusion of endometriosis were included as control women. Pregnancy at the time of the study as well as breast feeding were exclusion criteria for all participants. In both groups, only participants who answered a minimum of 80% of questions regarding past menstrual experiences were included. Finally, participants from the endometriosis group were matched to participants from the control group for age (±3years).

### Questionnaire

A structured self-administered questionnaire was developed by specialists in endometriosis and gyneco-psychosomatics from the universities of Zurich and Berlin as well as by the governing body of endometriosis support groups (1, 28). The questionnaire included 12 sections on basic socio-epidemiological data (Table 1), different areas of health and quality of life. For this study, general information and data on menarche, menstrual experiences, and endometriosis characteristics were evaluated. The study was registered on ClinicalTrials.gov (NCT 02511626), where further information about the questionnaire can be found. Disease characteristics, i.e., time since occurrence of first endometriosis related symptoms as perceived by the study participant, time interval between 1st symptom and 1st visit at gynecologist, and number of endometriosis-related surgical interventions were investigated by single choice questions with answer possibilities as shown in Table 2. Retrospective information on age at menarche, memories associated with first menstruation, mothers’ perceived attitude towards menstruation, and dysmenorrhea in mothers was collected via single choice questions with preselected answer possibilities, as shown in Table 3. For the variable “personal history of dysmenorrhea,” as shown in Table 3, participants were asked if they ever experienced strong discomfort during their menstruation (“yes currently,” “yes but not currently,” or “never”). Additionally, women who had experienced dysmenorrhea were asked about the onset of dysmenorrhea (“at menarche,” “2–3 years after menarche,” “>3 years after menarche,” or “unknown”).

**Table 1:** Participant characteristics.

	Women with endometriosis (*N* = 563)	Control women (*N* = 563)	*p*-value*
Age (mean ± SD in years)	37.99	37.53	0.314
Nationality (N/%)	562	561	0.068
Swiss/German/Austrian	504 (89.68%)	483 (86.10%)	
Other (non-specified)	58 (10.32%)	78 (13.90%)	
Educational level (N/%)	553	557	0.241
Mandatory school	26 (4.70%)	23 (4.13%)	
Secondary school/ Intermediate cchool certificate	117 (21.16%)	140 (25.13%)	
Apprenticeship/ Vocational diploma	215 (38.88%)	198 (35.55%)	
High school Diploma/University	195 (35.26%)	196 (35.19%)	
Parity^a^ (N/%)	520	534	**<0.001**
0	359 (69.04%)	263 (49.25%)	
1	93 (16.82%)	86 (16.10%)	
2	54 (10.38%)	134 (25.10%)	
≥3	14 (2.69%)	51 (9.55%)	

The bold *p*-values indicate the statistical significance in the bivariate analysis, significant *p*-values are Bonferroni corrected *α* = 0.01.

* Mann-Withney-U test for age, Pearson chi-squared test for other variables.

^a^ Parity defined as pregnancies carried out >24 weeks of gestational age.

SD, Standard deviation.

**Table 2:** Disease characteristics in women with endometriosis.

	Women with endometriosis (*N* = 563)
Time since occurrence of first endometriosis related symptoms (N/%)	528
<1 year	28 (5.30%)
1 year	25 (4.44%)
2–5 years	150 (26.64%)
6–10 years	108 (20.45%)
>10 years	217 (38.54%)
Time interval between 1st symptom and 1st visit at gynecologist (N/%)	487
1–4 weeks	104 (21.36%)
1–3 months	58 (11.91%)
3–6 months	70 (14.37%)
6–12 months	61 (12.53%)
>1 year	194 (39.84%)
Time between menarche and 1st endometriosis related surgery (mean ± SD in years)	14.03 ± 6.74
rASRM stage at latest surgery	561
I	99 (17.65%)
II	114 (20.32%)
III	166 (29.59%)
IV	182 (32.44%)
Number of endometriosis-related surgical interventions	526
1	281 (53.42%)
2	162 (30.80%)
≥3	83 (15.78%)

**Table 3:** Early menstrual experiences in women with endometriosis compared to control women.

	Women with Endometriosis (N = 563)	Control Women (N = 563)	p-value*
Somatic factors			
Age at menarche (N/%)	558	540	**0** **.** **004**
8–11 years	118 (21.15%)	71 (13.15%)	** **
12–15 years	393 (70.43%)	421 (77.96%)	** **
>15 years	47 (8.42%)	48 (8.89%)	** **
Cycle Length (N/%)	544	521	**<0** **.** **001**
Regular cycles, 4–6 weeks	276 (50.74%)	346 (66.41%)	
Regular cycles, <4 weeks	136 (25.00%)	91 (17.47%)	
Regular cycles, >6 weeks	15 (2.76%)	9 (1.73%)	
Irregular cycles	93 (17.10%)	62 (11.90%)	
Amenorrhea	24 (4.40%)	13 (2.50%)	
Personal history of dysmenorrhea			
Having had or having dysmenorrhea (N/%)	545	534	**<0** **.** **001** **^a^**
Never	35 (6.42%)	155 (29.03%)	** **
Yes, but now no dysmenorrhea	159 (29.17%)	219 (41.01%)	** **
Yes	351 (64.41%)	160 (29.96%)	** **
Onset of dysmenorrhea (N/%)	485	292	**<0** **.** **001** **^a^**
Yes, onset at menarche	219 (45.15%)	127 (43.49%)	
Yes, onset 2–3 years after menarche	92 (18.97%)	71 (24.32%)	
Yes, onset >3 years after menarche	174 (35.88%)	94 (32.19%)	
Memories associated with menarche (N/%)	554	533	**<0** **.** **001**
Only pleasant memories	42 (7.58%)	37 (6.94%)	** **
More pleasant than unpleasant memories	95 (17.15%)	146 (27.39%)	** **
More unpleasant than pleasant memories	221 (39.89%)	214 (40.15%)	** **
Only unpleasant memories	121 (21.84%)	38 (7.13%)	** **
No memories	75 (13.54%)	98 (18.39%)	** **
Dysmenorrhea in mother (N/%)	533	517	0.031
Very painful	106 (19.89%)	71 (13.73%)	
Somewhat painful	156 (29.27%)	137 (26.50%)	
Unknown	91 (17.07%)	97 (18.76%)	
Not painful	180 (33.77%)	212 (41.01%)	
Mothers’ attitude towards menstruation (N/%)	531	512	0.081
Rather positive	21 (3.95%)	17 (3.32%)	
Rather negative	196 (36.91%)	210 (41.02%)	
Unknown	114 (21.47%)	76 (14.84%)	
Neutral	200 (37.66%)	209 (40.82%)	

Data presented as *N* (%) using Mann-Withney-*U* test for age at menarche, Pearson chi-squared and Fisher's exact test for other variables.

^a^ Statistical significance remained after controlling for age and parity on bivariate logistic regression analysis.

* The bold *p*-values indicate the statistical significance in the bivariate analysis, significant *p*-values are Bonferroni corrected α = 0.01.

Irregularity of cycles is known to be associated with a 2-fold and short cycle lengths with a 1 to 1.5-fold increased risk for endometriosis, while long cycle lengths show 32% lower odds for the disease (6, 12, 30). Therefore, women were asked to report cycle length (“regular cycles of >6 weeks,” “regular cycles of 4–6 weeks,” “regular cycles <4 weeks,” “irregular cycles,” or “no menstruation”).

### Ethical approval

Local ethical review committees in Switzerland, Austria, and Germany provided ethical approval. The study was conducted according to the guidelines of the World Medical Association Declaration of Helsinki 1964, updated in October 2013.

### Statistical analysis

We performed bivariate analysis to assess differences in group characteristics between women with endometriosis and control women by applying the Pearson chi-squared test and the Mann-Whitney-U test. Binary logistic regression analysis was to identify reliable predictors for development and diagnosis of endometriosis. Before performing the regression analyses, variables were evaluated for multicollinearity. Pearson correlations <0.06, tolerance >0.75 and Variance Inflation Factor (VIF) <1.33 confirmed lack of multicollinearity. The significance threshold *α* was set to 0.05. We used the Bonferroni correction for multiple comparisons in all regression models. Adjusted odds ratios and their 95% CI were calculated for those factors who remained statistically significant in the bivariate logistic regression analysis. All statistical analyses were conducted using the R-Software, Version 4.1.2.

## Results

Our analysis included 1,128 women aged between 19 and 59 years, 563 of which presented with endometriosis. Further participant characteristics are shown in Table 1. Table 2 summarizes disease characteristics and reactions to disease symptoms in women with endometriosis. Results of the analysis of associations between early menstrual experiences and the development of endometriosis are presented in Table 3. Results of bivariate analysis will be presented first. Age at menarche, short cycles and personal history of dysmenorrhea were significantly associated with endometriosis in the analyses of crude data. Women who experienced menarche at 8–11 years of age had a higher probability of getting diagnosed with endometriosis than women who experienced menarche at 12–15 years or at >15 years of age (*p* = 0.004). We found that women with endometriosis showed lower parity and were more often nulliparous (69.04%) than control women (49.25%). Irrespective of the time of onset, dysmenorrhea was significantly associated with endometriosis (*p* < 0.001). Women diagnosed with endometriosis were more likely to report unpleasant memories of menarche compared to control women (*p* < 0.001), but still around 25% of them reported only pleasant or mainly pleasant memories. Altogether, 33% of the 335 women reporting dysmenorrhea within the first 3 years following menarche did not perceive this as an early symptom and reported first endometriosis symptoms only more than 10 years after menarche. We noted a slightly higher percentage of dysmenorrhea in mothers when endometriosis was diagnosed in their daughters (49.5% vs. 26.2%), which was not statistically significant. Mothers' attitudes towards menstruation did not correlate with the occurrence of endometriosis in their daughters.

After adjustment for confounders and performance of bivariate logistic regression presence of dysmenorrhea in women correlated with a 5.74-fold risk to show endometriosis (OR 5.74, 95% CI 3.82–7.22) while none of the other factors had any statistically significant association. Onset of menstrual pain at menarche was associated with a 2.35 risk (OR 2.35, 95% CI 1.44–3.88) and dysmenorrhea presenting >3 years after menarche with a more than 3-fold (OR 3.41, 95% CI 2.09–5.64) increased probability of being diagnosed with endometriosis (Table 4).

**Table 4:** Regression analysis evaluating the association between occurrence of dysmenorrhea and onset of dysmenorrhea in women with endometriosis compared to control women.

	Women with dysmenorrhea (now or in the past) (*N* = 511)	Women without dysmenorrhea ever (*N* = 568)	*p*-value*	Adjusted Odds ratio	95%CI
Having had or having dysmenorrhea (N/%)	545	534	**<0.001**		
Never	35 (6.42%)	155 (29.03%)		0.17	0.06–0.45
Yes, but now no dysmenorrhea	159 (29.17%)	219 (41.01%)		0.26	0.09–0.67
Yes	351 (64.41%)	160 (29.96%)		5.74	3.82–7.22
Onset of dysmenorrhea (N/%)	485	292	**<0.001**		
Yes, onset at menarche	219 (45.15%)	127 (43.49%)		2.35	1.44–3.88
Yes, onset 2–3 years after menarche	92 (18.97%)	71 (24.32%)		2.38	1.41–4.07
Yes, onset >3 years after menarche	174 (35.88%)	94 (32.19%)		3.41	2.09–5.64

* The bold *p*-values indicate the statistical significance in the bivariate analysis, significant *p*-values are Bonferroni corrected *α* = 0.01.

Furthermore, a comparison between women with and without dysmenorrhea was done, irrespective of diagnosis of endometriosis. Women with dysmenorrhea showed a tendency to having an earlier age (8–11 years) at menarche with onset of dysmenorrhea at menarche compared to women without menstrual pain (47.34% vs. 38.85%). Memory of menarche was reported more frequently as negative when accompanied by dysmenorrhea (*p* < 0.001, Table 5). In participants with (rather) negative memories of menarche, 48.42% had experienced dysmenorrhea at menarche in contrast to only 16.77% in women with (rather) positive memories of menarche. Study participants with dysmenorrhea more frequently had mothers who were also affected by dysmenorrhea than those without dysmenorrhea (54.26%/ 36.68%, *p* < 0.001, Table 4). Perceived attitudes of mothers towards menstruation just slightly differed between women with and without dysmenorrhea.

**Table 5:** Investigation of mothers’ and daughters’ experience and perception of menstruation.

	Women with dysmenorrhea (now or in the past) (*N* = 511)	Women without dysmenorrhea ever (*N* = 568)	*p*-value*
Age at menarche	510	568	**<0.001**
8–11 years	103 (20.20%)	83 (14.61%)	
12–15 years	368 (72.16%)	432 (76.06%)	
>15 years	39 (5.64%)	53 (9.33%)	
Onset of dysmenorrhea	507	260	**<0.001**
Yes, onset at menarche	240 (47.34%)	*101* (*38.85%)*	
Yes, onset 2–3 years after menarche	95 (18.75%)	65 (25%)	
Yes, onset >3 years after menarche	172 (33.92%)	94 (36.15%)	
Memories associated with menarche	506	562	**<0.001**
Only pleasant memories	27 (5.34%)	50 (8.90%)	
More pleasant than unpleasant memories	93 (18.38%)	146 (25.98%)	
More unpleasant than pleasant memories	211 (41.70%)	217 (38.61%)	
Only unpleasant memories	107 (21.15%)	47 (8.36%)	
No memories	*68* (*13.44%)*	*102* (*18.15%)*	
Dysmenorrhea in mother	481	548	**<0.001**
Very painful	115 (23.91%)	61 (11.13%)	
Somewhat painful	146 (30.35%)	140 (25.55%)	
Unknown	75 (15.59%)	109 (19.89%)	
Not painful	145 (30.15%)	238 (43.43%)	
Mothers’ attitude towards menstruation	479	543	**<0.001**
Rather positive	21 (4.38%)	16 (2.95%)	
Rather negative	185 (38.62%)	216 (39.78%)	
Unknown	112 (23.38%)	75 (13.81%)	
Neutral	161 (33.61%)	236 (43.46%)	

Data presented as *n* (%) using Mann-Withney-*U* test for age at menarche, Pearson chi-squared and Fisher's exact test for other variables.

*The bold *p*-values indicate the statistical significance in the bivariate analysis, significant *p*-values are Bonferroni corrected *α* = 0.01.

## Discussion

In our study, dysmenorrhea at menarche or thereafter was strongly associated with the development of endometriosis, which agrees with results from other studies (21, 31, 32). Our findings showed a 5.7-fold risk for a diagnosis of endometriosis in case of dysmenorrhea at or after menarche, which suggests that endometriosis should be carefully evaluated in adolescent girls with dysmenorrhea.

Young age at menarche and a short cycle length were also associated with diagnosis of endometriosis supporting previously published literature (33, 34), while parity (35, 36) was associated with a decreased risk. This is in line with a long-appreciated concept of endometriosis as an estrogen-dependent disorder and is well supported by molecular evidence (33) as the development and growth of endometriosis is closely related to steroid metabolism (35). Although the exact timing of menarche cannot be predicted in individual cases, it is known that maturity of the GnRH pulse generator has a leading role, while further factors such as ethnicity or body weight influence timing of menarche (37). Women with early menarche are characterized by an earlier exposure to estrogens, i.e., circulating estradiol and estrone levels, which stimulate ectopic and eutopic endometrial tissue (38). 17β-Estradiol is known to be a key hormone for the growth and persistence of endometriotic tissue as well as related inflammation and pain with a particular relevance of local concentrations (39).

Early menstruation is very frequently associated with dysmenorrhea, typically with onset up to 12 months after menarche (40, 41). As the menstrual cycle matures within 1 to 4 years (42), especially dysmenorrhea persisting thereafter should result in considering the presence of endometriosis. Our data showed a further increase in risk of endometriosis if dysmenorrhea occurs >3 years after menarche being in line with the fact that endometriosis is the most common cause of secondary dysmenorrhea (43). Pelvic anomalies or high uterine contractibility, which may contribute to dysmenorrhea at menarche on the one hand and could on the other hand be predisposing factors for later endometriosis (18, 19, 21), should be investigated carefully. Recently, saliva tests to detect endometriosis have become available, so that noninvasive evaluation of endometriosis has become possible and unnecessary surgery in most female adolescents who do not suffer from endometriosis can be avoided (44).

Interestingly, in study participants, dysmenorrhea at or following menarche was frequently not perceived as a symptom of endometriosis. Despite experiencing early dysmenorrhea, 33% of the women reported first endometriosis symptoms more than 10 years later. A potential reason for recognizing dysmenorrhea as a symptom related to endometriosis very late might be that endometriosis indeed developed at a later point in life and pain characteristics changed. Dysmenorrhea in endometriosis is often reported as both, characteristic and particularly severe pain and we have no information on the exact onset of disease in our study population. Information about presence and distribution of endometriotic lesions at time of menarche and at time of subjective onset of symptoms is limited since surgical exploration was performed many years later in most participants. It is also very likely that even severe dysmenorrhea is still perceived as a normal feature of menstruation by many women. Therefore, health care professionals and women themselves often do not evaluate causes as they should. As awareness that endometriosis may already be present in adolescents is still increasing (45, 46), adolescent girls as well as doctors are likely not aware of the significance of dysmenorrhea as a key symptom of endometriosis in this age group. Subsequently, the origins of early dysmenorrhea are often not investigated; and need for treatment is not ascertained. The identification of a possible early manifestation of endometriosis is further hampered by the fact that adolescents present different symptoms from adults, i.e., they experience noncyclic and cyclic pelvic pain more frequently, more severe pain, and pain accompanied by nausea (15, 47). However, a prevalence of endometriosis in 64% of adolescents with chronic pelvic pain (9) suggests that endometriosis is underdiagnosed in adolescents. Early investigation of endometriosis is further supported by the long diagnostic delay of endometriosis, i.e., 8–10 years even in countries with a well-established health care system (48, 49), which is partly due to the requirement of surgery for confirmation of endometriosis during the time of our study. In line with these results, more than 40% of our study participants consulted their family doctor or their gynecologist more than 1 year after perception of symptoms potentially related to endometriosis. In addition, the mean duration between menarche and surgical confirmation of endometriosis in women experiencing dysmenorrhea either directly or within 3 years after menarche was 14.03 ± 6.74 years, i.e., not only the first step towards diagnosis was taken very late, but also the step allowing reliable diagnosis. Since endometriosis frequently impairs fertility, early diagnosis is very important for family planning. Especially in advanced stages of endometriosis fertility may be strongly impaired and often requires artificial reproduction techniques, where still success rates may be limited (12, 43). Early diagnosis of endometriosis does not only allow early treatment to avoid a negative impact on fertility but also better timing of family planning or even egg freezing before a significant amount of ovarian tissue is lost to surgery, so that a combined negative effect of endometriosis and age-related limitations of fertility can be prevented.

We are therefore confident that our findings help to improve diagnostic and therapeutic management of endometriosis in adolescents. Ultrasound techniques to diagnose endometriosis are continuously improving, and hormonal treatments provide valuable resources to identify causes of dysmenorrhea (15, 16) as alternatives to laparoscopy in adolescents, for whom indications for surgery should be taken particularly cautiously. Recently, saliva tests to detect endometriosis have become available, so that noninvasive evaluation of endometriosis has become possible (44). The evaluation of dysmenorrhea within the patient's history has already been shown to be helpful for early detection of endometriosis (31). Short questionnaires with preselected answers as used in our study are appreciated by patients, initiate discussion of symptoms with doctors (31), and might therefore facilitate pinpointing a risk for endometriosis in adolescent girls.

Early menstrual experiences are likely the result of symptoms accompanying menstruation and the attitude towards menstruation influenced by mothers. Our data show a significant association between mothers' history of dysmenorrhea occurrence of dysmenorrhea in their daughters, which agrees with previous literature (17). Mothers' perceived negative attitude towards menstruation was not associated with endometriosis or with occurrence of dysmenorrhea in in their daughters. Our data support previous findings (22–24) indicating that mothers' experiences with menstruation might be very similar like their daughters' experiences, mainly shaped by similar experiences of dysmenorrhea probably also resulting from endometriosis. Since first-degree relatives of women with endometriosis have a 3- to 9-fold increased risk to develop the disease (14, 32), these associations likely result from hereditary factors. Although our data showed no increase of endometriosis in daughters if their mother have suffered from dysmenorrhea, it is highly possible, that dysmenorrhea in the family history may help to improve early diagnosis of endometriosis. Unfortunately, we had no information about diagnosis of endometriosis in mothers, which makes families more aware of endometriosis as a cause of dysmenorrhea and consequently facilitates early diagnosis. Likely recall bias in terms of remembering dysmenorrhea in mothers better, when there are own experiences of dysmenorrhea will have influenced our results, i.e., have caused underreporting of dysmenorrhea in mothers. Also, not all study participants might have been aware of their mothers' dysmenorrhea.

We concluded that there is a significant association between early age at menarche and the development of endometriosis, which is in line with some (12–14) but not all previous studies (20, 44). However, while women who had their menarche at 8–11 years were more likely to develop endometriosis, late menarche did not prove to be a protective factor. Our data strongly indicated that dysmenorrhea clearly is the most important factor in risk estimation for endometriosis.

## Strengths and limitations

To our best knowledge, this study provides the largest case-control matched sample investigating the association between early menstrual experiences and endometriosis, controlled for influences of mothers' perceived attitudes and experiences with menstruation. Endometriosis was surgically verified, i.e., diagnostic accuracy is very high. However, as women with endometriosis may also be symptom free, some affected women might have been included as controls, which would lead to the underestimation of effects. Women were recruited in university hospitals, districts hospital and private offices to aim for a representative sample, however a high proportion of women suffers from ASRM stage IV endometriosis, so that generalization of data should be done on the background of the characteristics of the study group. The detailed self-structured questionnaire allowed to evaluate experiences going back many years, but recall and response bias, might have influenced results, although menarche is an important life event in women's lives, which facilitates adequate memorization. Especially knowledge of daughters about dysmenorrhea in their mothers might be inaccurate and superficial, but still in each group, endometriosis and control group around 70% of participants were able to answer this question properly.

An important limitation of this study is that we cannot differentiate between women who presented with dysmenorrhea because of current endometriotic lesions and women who reported dysmenorrhea and only later developed endometriotic lesions. As we have no information on pain treatment at the time of menarche and early menstruations, we cannot exclude that successful treatment of dysmenorrhea led to a later diagnosis of endometriosis. The lack of knowledge that endometriosis can be present already at menarche (45, 46), at the time our participants experienced their menarche, influenced diagnostic approaches.

## Implications and conclusion

Dysmenorrhea is a well-known phenomenon accompanying the maturing menstrual cycle and is strongly associated with a diagnosis of endometriosis. Negative menstrual experiences, most likely resulting from similar physical experiences such as dysmenorrhea, are often shared with mothers. Considering the high prevalence of endometriosis in adolescents with dysmenorrhea as well as the consequences of endometriosis on adolescents’ reproductive health, the causes of dysmenorrhea should be investigated very carefully, especially if it does not respond to medical management.

## Data Availability

The original contributions presented in the study are included in the article/Supplementary Material, further inquiries can be directed to the corresponding author.
